# MRI reconstruction with enhanced self-similarity using graph convolutional network

**DOI:** 10.1186/s12880-024-01297-2

**Published:** 2024-05-17

**Authors:** Qiaoyu Ma, Zongying Lai, Zi Wang, Yiran Qiu, Haotian Zhang, Xiaobo Qu

**Affiliations:** 1 School of Ocean Information Engineering, Jimei University, Xiamen, China; 2 Department of Electronic Science, Biomedical Intelligent Cloud R&D Center, Fujian Provincial Key Laboratory of Plasma and Magnetic Resonance, National Institute for Data Science in Health and Medicine, Xiamen University, Xiamen, China

**Keywords:** Graph convolutional network, Deep learning, Fast magnetic resonance imaging, Image reconstruction

## Abstract

**Background:**

Recent Convolutional Neural Networks (CNNs) perform low-error reconstruction in fast Magnetic Resonance Imaging (MRI). Most of them convolve the image with kernels and successfully explore the local information. Nonetheless, the non-local image information, which is embedded among image patches relatively far from each other, may be lost due to the limitation of the receptive field of the convolution kernel. We aim to incorporate a graph to represent non-local information and improve the reconstructed images by using the Graph Convolutional Enhanced Self-Similarity (GCESS) network.

**Methods:**

First, the image is reconstructed into the graph to extract the non-local self-similarity in the image. Second, GCESS uses spatial convolution and graph convolution to process the information in the image, so that local and non-local information can be effectively utilized. The network strengthens the non-local similarity between similar image patches while reconstructing images, making the reconstruction of structure more reliable.

**Results:**

Experimental results on in vivo knee and brain data demonstrate that the proposed method achieves better artifact suppression and detail preservation than state-of-the-art methods, both visually and quantitatively. Under 1D Cartesian sampling with 4 × acceleration (AF = 4), the PSNR of knee data reached 34.19 dB, 1.05 dB higher than that of the compared methods; the SSIM achieved 0.8994, 2% higher than the compared methods. Similar results were obtained for the reconstructed images under other sampling templates as demonstrated in our experiment.

**Conclusions:**

The proposed method successfully constructs a hybrid graph convolution and spatial convolution network to reconstruct images. This method, through its training process, amplifies the non-local self-similarities, significantly benefiting the structural integrity of the reconstructed images. Experiments demonstrate that the proposed method outperforms the state-of-the-art reconstruction method in suppressing artifacts, as well as in preserving image details.

## Background

Magnetic Resonance Imaging (MRI) is an indispensable non-radiative medical imaging technology with excellent tissue resolution. However, its practical application is constrained by inherently long data acquisition times, a limitation that has sparked considerable interest in the acceleration technique [1]. Among these, parallel imaging [2] and undersampling [1] strategies have been prominently pursued to expedite MRI data acquisition. While undersampling is a viable approach to speed up data acquisition, it tends to introduce artifacts into the images. Compressed Sensing (CS) [1] has emerged as a powerful approach to address these artifacts by leveraging image sparsity in a transform domain, especially under an adaptively trained sparse representation [3, 4]. Additionally, in pursuit of enhanced sparsity, several methodologies have been explored to incorporate prior knowledge from similar image patches [5–7]. For instance, the non-local total variation (NLTV) [7] explores the similarity by measuring the Gaussian distance of image patches and using the weighted total variation to sparsity image pix. The patch-based non-local operator (PANO) [5] learns similarity through grouping similar patches of a pre-reconstruction of the target image and sparsify grouped patches with 3D wavelets. The graph-based redundant wavelet transform (GBRWT) [6], by viewing each patch as a node on a graph and the difference of image patch as the edge, the similarity is denoted as a shortest travel over the graph. The order of traveling each node (image patch) is also the order of sorting image pixels. Then, 1D wavelets is used to sparsify the sorted image pixels. These advanced techniques rely on a pre-reconstructed image to ascertain the similarity, thus the reconstruction may be unsatisfactory if the pre-reconstruction is not good under high acceleration factor of fast sampling [8]. This emphasizes the ongoing need for improvements in MRI reconstruction methodologies to achieve high-quality imaging efficiently.

Inspired by deep learning [9–11], initial approaches to deep learning-based MRI reconstruction predominantly employed Convolutional Neural Networks (CNNs) to carry out the reconstruction process [12–26]. These early deep learning models, leveraging convolutional kernels learned from MRI image datasets, excelled in capturing local spatial details within the grid-like structure of images, thereby demonstrating a robust capability for feature representation. Recent innovations have further expanded these capabilities. For instance, the SOGAN [27] framework introduces compact attention maps to encapsulate long-range contextual information across both vertical and horizontal planes, thereby significantly elevating the quality of MRI reconstruction. Similarly, DONet [28] explores multi-scale spatial-frequency features, while MD-Recon-Net [29] enhances reconstruction efficiency by operating in parallel across k-space and spatial domains. Additionally, DC-WCNN [30] introduces the use of wavelet transform as an alternative to traditional pooling layers to extract multiple information in MRI images. These addressed the limitations of earlier models that primarily focused on local features. However, these methods often overlooked the potential of non-local self-similarity within images.

The emergence of graph structures to encapsulate adjacency relationships presents a novel way to model non-local interactions within data [31–34]. However, conventional Convolutional Neural Networks (CNNs) are not inherently equipped to leverage these graph structure. The Similarity-Guided Graph Neural Network (SGGNN) [35] creates a graph to represent the pairwise image relationships and utilized the similarity between images to learn the edge weights with rich labels of gallery instance pairs directly.

Building on these insights, we propose a Graph Convolution network with Enhanced Self-Similarity (GCESS) to reconstruct MRI images from undersampled k-space data. This method leverages aggregating similar image patches as prior information and employs graph convolution to filter these sets of similar patches. Accurately estimating self-similarity is crucial for the effectiveness of graph convolutional neural networks. Ideally, optimal self-similarity should be estimated on a fully sampled image, which is not available in fast MRI. To alleviate this problem, we propose to estimate self-similarity from a pre-reconstructed image obtained by a conventional reconstruction method SPIRiT [36]. During the training phase, graph filters undergo refinement, enhancing the self-similarity within the images by restoring the graph nodes. Furthermore, a spatial convolution process is incorporated to simultaneously leverage local and non-local information for more effective image reconstruction. This dual approach ensures a comprehensive utilization of available data, optimizing the reconstruction process. Our main contributions are: 1) The non-local self-similarity guided graph convolution is combined with local spatial convolution for improved MRI reconstructions. 2) Comprehensive evaluations on in vivo datasets, illustrating that GCESS surpasses existing state-of-the-art methods in visual and quantitative metrics, particularly in reducing artifacts and enhancing detail preservation. The GCN-Unet framework [37] has been suggested in our previous work as a solution to the over-smoothing issue inherent in Graph Convolutional Networks (GCN), specifically for processing non-local information in MRI image reconstruction. However, it did not thoroughly analyze the graph representation of non-local self-similarity. And by combining non-local and local information, a different network structure is proposed in the proposed method.

## Methods

In this section, we introduce the entire implementation process of Graph Convolution network with Enhanced Self-Similarity (GCESS) in detail. The GCESS network innovatively integrates graph convolution with spatial convolution, leveraging both non-local self-similarities and local information to enhance MRI image reconstruction. Specifically, we employ a patch graph to capture non-local information, connecting MRI image patches through nodes that represent vectorized patches, with the weight of edges is the differences between these patches. This phase initially enhances the self-similarity in the MRI images. Following this, the network harnesses both non-local and local information during training to reconstruct image patches. These reconstructed patches exhibit improved structural features, further amplifying the similarity weight between similar image patches, allowing for better restoration of the image structure. Before introducing GCESS network, we review the basic MRI reconstruction model [38].

When an image is sufficiently sparse in the transform domain, the theory of CS [1] enables accurate image recovery from limited measurement data. The basic MRI imaging model in CS can be written as [38]:

1 $$\mathop {\min }\limits_{x} \lambda \mathop \sum \limits_{j = 1}^{J} \left\| {{\varvec{y}}_{j} - {\varvec{F}}_{u} {\varvec{C}}_{j} {\varvec{x}}} \right\|_{2}^{2} + \mathcal R\left(\boldsymbol x\right),$$

where ${\varvec{x}} \in {\mathbb{C}}^{M \times N}$ is the reconstructed image, ${\varvec{y}}_{j} \in {\mathbb{C}}^{M \times N}$ is the undersampled k-space data acquired from the $j^{th}$ coil, ${\varvec{C}}_{j}$ is the sensitivity map of $j^{th}$ coil, ${\varvec{F}}_{u} = {\varvec{UF}} \in {\mathbb{C}}^{M \times N}$ denotes the undersampled Fourier transform operator ($M < N$). $\left\| \cdot \right\|_{2}$ stands for $l_{2}$ norm which enforces the fidelity of the reconstruction to the measured k-space data. $\lambda$ is a weight to balance the data consistence and regularization term. $\mathcal R\left(\boldsymbol x\right)$ in the context of Deep Learning-based Compressed Sensing MRI encapsulates the model’s assumptions about the underlying image characteristics, such as sparsity in certain transforms and its proximity to outcomes from deep learning reconstructions. This methodology is formalized as follows:

2 $$\mathop {\min }\limits_{x} \lambda \mathop \sum \limits_{j = 1}^{J} \left\| {{\varvec{y}}_{j} - {\varvec{F}}_{u} {\varvec{C}}_{j} {\varvec{x}}} \right\|_{2}^{2} + \left\| {f_{nn} \left( {{\varvec{z}}|{\varvec{\theta}}} \right) - {\varvec{x}}} \right\|_{2}^{2}$$

where $f_{nn} \left( \cdot \right)$ symbolizes the neural network model parameterized by ${{\varvec{\uptheta}}}$. ${\varvec{z}}$ and $f_{nn} \left( {\left. {\varvec{z}} \right|{{\varvec{\uptheta}}}} \right)$ denote the input and output of model respectively. The input can be either $\varvec{y}$ (the undersampled data) or ${\varvec{x}}_{u}$(zero-filling solution reconstructed from $\varvec{y}$), and the output denotes the predicted reconstruction result. The essence of this approach lies in the network architecture design within the framework, aiming to either augment or completely substitute the energy minimization process traditionally used in MRI reconstruction with the neural network’s training process. This work introduces a deep network regularization term that incorporates both local and non-local information. We start from the representation of non-local self-similarity in the following section.

### Graph representation of self-similarities

The local and non-local information is crucial to be constrained for MRI reconstructions. Local information is processed using local spatial convolution, consistent with the approach of most existing methods [12–20]. For non-local information, this study constructs a patch graph to harness non-local information through self-similarity to establish a graph convolutional network. In this framework, graph nodes are vectorized image patches while the weights within patch graph signify the similarities between these patches. Through graph network learning, this approach capitalizes on the non-local self-similarity in the image for the reconstruction of patches.

**Fig. 1:**
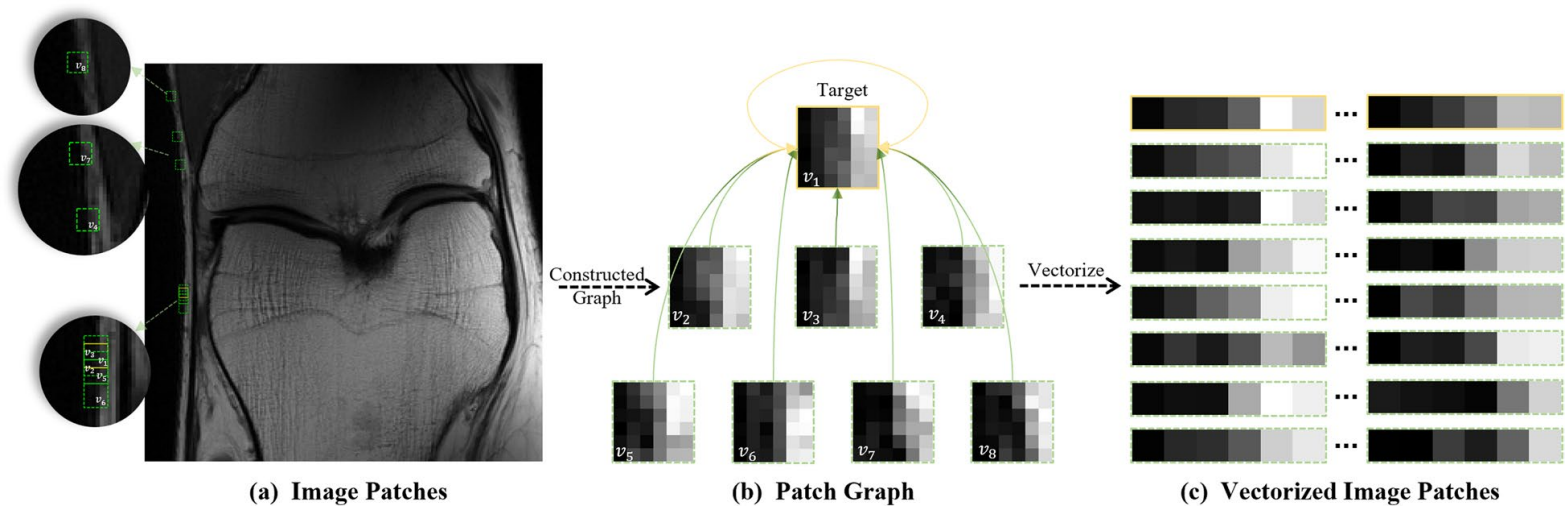
The whole process of constructing a graph from an image. Representation of non-local self-similarity with a patch graph of fully sampled MRI image. The image patch bounded by the solid yellow line is set as the target image patch. Similar image patches are represented by green dotted lines distributed in the image. **a** The eight most similar image patches are found in the global image. **b** Graph is constituted with a similar patch found in (**a**). **c** Vectorized image patches (nodes)

Specifically, for every node (target image patch) in the graph, we search the eight most similar image patches (including self-connection) as the connected nodes. The patch graph is set as ${\mathcal{G} }({\mathcal{V}},{\mathcal{E}})$ with $N$ nodes $v_{i} \in {\mathcal{V}}$ and edges $\left( {v_{i} ,v_{j} } \right) \in {\mathcal{E}}$, $i,j = 1,2, \cdots ,N$. Figure 1a-b demonstrate that one target image patch (node $v_{1}$) connects with its most similar patches. The weight (Euclidean distance [39, 40] represent the similarity scores between $v_{i}$ and $v_{j}$) on the edges $\left( {v_{i} ,v_{j} } \right) \in {\mathcal{E}}$ constitute different adjacency matrix ${\hat{\varvec{A}}} \in {\varvec{R}}^{N \times N}$. Consequently, image patches with more similarities, which are not adjoined in the grid-like images, are connected by edges with patch similarity scores in the graph. These similarities scores will be further refined during network training to bolster the efficiency of graph convolutional neural networks in MRI reconstructions.

To emphasizes the pairwise relationships between a node and the information from its adjacent nodes, the Gaussian function is employed to weight all Euclidean distances [41]:

3 $${\varvec{A}}_{ij} = \exp \left( { - \frac{{\left\| {v_{i} - v_{j} } \right\|_{2}^{2} }}{{\sigma \left({\mathcal{V}} \right)^{2} }}} \right),$$

where $\sigma \left( {\mathcal{V}} \right)$ is the standard deviation of the nodes. The Gaussian function possesses normalization ability for weights which can prevent the filter from updating unnecessary dimensional gaps to reduce computational complexity. This mechanism effectively emphasizes the most critical weights, ensuring focus is maintained on the most pertinent connections. Obviously, when employing the Gaussian function, the weight of the self-connected edge of target patch is 1.

**Fig. 2:**
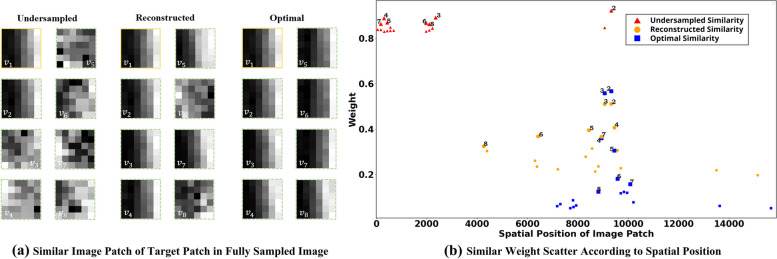
Selection of most seven similar connected patches in different reference image. **a** The target patch v1 and its seven most similar patches v2-v8 in undersampled, reconstructed and optimal similarity. To better see the difference, image patches are selected from fully sampled image. **b** Similarity weights to the target patch in different references (self-connection is excluded). The seven most similar nodes emphasize with larger dots compared with others. Note: Optimal similarity means that weights are calculated from fully sampled image. Undersampled similarity means that weights are calculated from an undersampled image. Reconstructed similarity means that weights are calculated from image reconstructed by conventional parallel MRI methods

A graph representing self-similarity is summarized in the Fig. 1. These interconnected patches share information, and can be aggregated to reconstruct the target patch. The selection of connected patches is influenced by the reference image. Reference images containing significant artifacts can lead to selections that do not accurately reflect true similar relationships. Figure 2 demonstrates the comparison of undersampled similarity, reconstructed similarity and optimal similarity. Here, undersampled similarity means that similarity weights are calculated from undersampled image, reconstructed similarity means that similarity weights are calculated from image reconstructed by a conventional MRI reconstruction method, i.e. iterative Self-consistent Parallel Imaging Reconstruction (SPIRiT) [36], and optimal similarity means that similarity weights are calculated from fully sampled image. The adjacency weight, as shown in Fig. 2, is annotated on the graphic according to spatial position of image patches. This illustration reveals that the similar relationship in the undersampled image is inconsistent with the optimal scenario. The similarity weight derived from a pre-reconstructed image aim to align more closely with the optimal similarity, as depicted in Fig. 2b. Such similarity relationships are pivotal for graph convolution which will be leveraged to train graph convolution to facilitate the target image reconstruction. The impact of similarity on the reconstruction results will be discussed in the subsequent section. We introduce how network utilizes the generated graph structure to reconstruct MRI images in following section.

### Graph convolution with enhanced self-similarity

The deep network regularization of this paper integrates a graph convolution learning process leveraging non-local similarity. This method enhances non-local patch-pair similarities which then aids in the reconstruction of the nodes. Initially, the feature of the nodes in the graph are represented as vectorized image patches. The adjacency matrix ${\varvec{A}}$ corresponds to the measured similarity of each patch pair. In the graph, each node is assigned a single degree of connection. The degree ${\varvec{D}}_{ii} = \sum\limits_{j}^{N} {{\varvec{A}}_{ij} }$ $\left( {i,j = 1,2, \cdots ,N} \right)$ refers to the total influence of the* i*-node across all nodes within the graph, and node degrees form a diagonal degree matrix, i.e. ${\varvec{D}} = diag\left( {{\varvec{D}}_{ii} } \right)$. The graph Laplacian is normalized ${\varvec{L}} = {\varvec{I}}_{N} - {\varvec{D}}^{{ - \frac{1}{2}}} {\varvec{AD}}^{{ - \frac{1}{2}}} = \varvec{U\Lambda U}^{T}$, where $\varvec{U}$ represents the matrix of eigenvectors and ${\varvec{\varLambda}}$ is a diagonal matrix of eigenvalues of the normalized graph Laplacian. This framework allows the spectral graph convolution [32] to analyze the non-local similarities represented in the graph structure,

4 $${\varvec{g}}_{\theta } * {\varvec{M}} = {\varvec{Ug}}_{\theta } {\varvec{U}}^{T} {\varvec{M}},$$

where ${\varvec{M}} \in {\varvec{R}}^{N \times C}$ is a matrix of node features stacked by row. ${\varvec{U}}^{T} {\varvec{M}}$ is the Fourier transform of $\varvec{M}$. ${\mathbf{g}}_{\theta } = diag\left( \theta \right)$ is a spectral filter parameterized by ${{\varvec{\uptheta}}} \in {\mathbf{R}}^{N}$. Without loss of generality, scalar nodes are used instead to explain the proposed graph convolution process, and thus ${\varvec{m}} \in {\varvec{R}}^{N}$ is used instead of $\varvec{M}$ in the following explanation. The ${\varvec{g}}_{\theta }$ can be further understood as a function of the eigenvalues of $\varvec{L}$, i.e. $g_{\theta } \left( {{\varvec{\Lambda}}} \right)$.

The process of eigen-decomposition is characterized by low efficiency and high computational complexity. To circumvent this problem, it was suggested by Hammond et al. [42] that $g_{\theta } \left( {{\varvec{\Lambda}}} \right)$ can be well-approximated by a truncated expansion in terms of Chebyshev polynomials $T_{k} \left( {{\varvec{\Lambda}}} \right)$. The independent variables of $T_{k} \left( {{\varvec{\Lambda}}} \right)$ are required to be varied within the range [-1, 1]. In this case, the eigenvalues ${{\varvec{\Lambda}}}$ are rescaled as ${\tilde{\mathbf{\Lambda }}} = \left( {2/\lambda_{\max } } \right){{\varvec{\Lambda}}} - {\varvec{I}}_{N}$, where $\lambda_{\max }$ denotes the largest eigenvalue of ${\varvec{L}}$. $\lambda_{\max }$ approximately equals to 2, which can be expected that neural network parameters will adapt to this change in scale during training. Thus, the graph convolution with Chebyshev polynomial can be reformulated as

5 $${\varvec g_\theta } * \varvec m = \varvec U{\varvec g_{\theta^\prime}}\left( \tilde {\varvec \Lambda } \right){\varvec U^T}\varvec m \approx \sum\limits_{k = 0}^1 {\theta_k^\prime} {T_k}\left( \tilde {\varvec L} \right)\varvec m = \theta_0^\prime \varvec m + \theta_1^\prime \tilde { \varvec L}\varvec m,$$

with rescaled normalized graph Laplacian $\tilde{\varvec L} = \left( {2/\lambda_{\max } } \right){\varvec{L}} - {\varvec{I}}_{N}$. $\theta^\prime_{0}$ and $\theta^\prime_{1}$ are coefficients of Chebyshev polynomials. The 1st order Chebyshev polynomials are defined as $T_{1} \left( {\tilde{ \varvec {L}}} \right) = 1 + \tilde{\varvec {L}}$. By assigning identical values to these parameters, namely setting $\theta^\prime = \theta^\prime_{0} = - \theta^\prime_{1}$, the Eq. (5) is further simplified as

6 $${\varvec{g}}_{\theta } * {\varvec{m}} = \theta^\prime_{0} {\varvec{m}} - \theta^\prime_{1} {\varvec{D}}^{{ - \frac{1}{2}}} {\varvec{AD}}^{{ - \frac{1}{2}}} {\varvec{m}} = \theta^\prime \left( {{\varvec{I}}_{N} + {\varvec{D}}^{{ - \frac{1}{2}}} {\varvec{AD}}^{{ - \frac{1}{2}}} } \right){\varvec{m}}.$$

The eigenvalue of ${\varvec{I}}_{N} + {\varvec{D}}^{{ - \frac{1}{2}}} {\varvec{AD}}^{{ - \frac{1}{2}}}$ is more than 1. Therefore, repeating this operation in the deep learning model will lead to numerical instability and explosion gradient. To alleviate these problems, ${\varvec{I}}_{N} + {\varvec{D}}^{{ - \frac{1}{2}}} {\varvec{AD}}^{{ - \frac{1}{2}}}$ is renormalized as $\tilde {\varvec D}^{{ - \frac{1}{2}}} {\tilde {\varvec A}\tilde{\varvec D}}^{{ - \frac{1}{2}}}$. $\tilde{\user2{A}} = {\varvec{A}} + {\varvec{I}}_{N}$ is the adjacent matrix of graph that each node has self-connecting edge and $\tilde{\varvec {D}}_{ii} = \sum\limits_{j}^{N} {\tilde{\varvec {A}}_{ij} }$ is the degree of *i*-th node. Then the graph convolution becomes

7 $${\varvec{g}}_{\theta } * {\varvec{m}} \approx \theta^\prime \tilde{\varvec {D}}^{{ - \frac{1}{2}}} {\tilde {\varvec A}\tilde{\varvec D}}^{{ - \frac{1}{2}}} {\varvec{m}}.$$

This equation realizes node feature filtering guided by similarity weight with a spectral graph convolution operation. Then the* i*-th node feature can be reformulated as:

8 $$\theta \left( {\tilde{\varvec {D}}^{{ - \frac{1}{2}}} {\tilde{\varvec A}\tilde{\varvec D}}^{{ - \frac{1}{2}}} {\varvec{m}}} \right)_{i} = \theta \sum\limits_{j}^{N} {\frac{{\tilde{\varvec{A}}_{ij} {\varvec{m}}_{j.} }}{{\sqrt {\tilde{\varvec{D}}_{ii} \tilde{\varvec{D}}_{jj} } }},}$$

where $\tilde{\varvec{D}}_{ii}$ denotes *i*-th node (target node) degree and $\tilde{\varvec{D}}_{ii}$ denotes j-th node degree in the graph. $\tilde{\varvec{A}}_{ij}$ is the similarity weights between i-th and j-th node. Node features are refined by fusing most similar connected nodes with a graph convolution process. The non-local information is aggregated by selecting the most similarity weight through the graph. The larger weight of $\tilde{\varvec{A}}_{ij}$ representing, the more similarities between nodes ($v_{i}$ and $v_{j}$), and the greater contribution can be obtained in target node reconstruction. To minimize the impact of unimportant weights, except for the most similar weights, others are set to zero.

Generalizing the graph filtering process to a signal $\varvec {M} \in \varvec {R}^{N \times C}$ with *C* input channels (a *C*-dimensional feature vector for every node):

9 $$\varvec Z = {\tilde {\varvec D}^{ - \frac{1}{2}}}{\tilde{\varvec A} \tilde{\varvec D}^{ - \frac{1}{2}}}\varvec M \varvec \Theta ,$$

where $\Theta \in {\varvec{R}}^{C \times F}$ is filter parameter and ${\varvec{Z}} \in {\varvec{R}}^{N \times F}$ is feature matrix after convolution. This is also in line with practical MRI reconstruction, where noise and artifacts usually contaminate image pixels. In this case, when patch nodes are used instead of scope pixel, edge weights calculation and the subsequent graph convolution will be insensitive to noise and artifacts. Then the aggregation of non-local information with self-similarity to reconstruct target image will be robust.

In the method described in this section, network training enables the graph convolution kernel to adjust its parameter weights, thereby managing the information transfer between the target image patch and similar image patches through connection edges. This process allows the target image patch to rapidly acquire information from highly similar image patches, leveraging the structural information of similar patches during the reconstruction. As a result, the structural information of each image patch is restored after reconstruction, further enhancing the similarity between the target image patch and its connected image patches. Moreover, due to the richer and more authentic graph structural information, the graph convolutional kernel can more effectively extract similarity information from the graph structure. Thus, both the reconstruction of image patches and the training of the graph convolution kernel mutually benefit. The proposed feature updating is intuitive since the rich non-local information with enhanced self-similarity are effectively exploited. Steering the refinement of node features with similarity weights paves the way for more precise feature reconstruction. It is worth noting that filter adaptively performs weighting with the most similarity in the graph to update target node features for reconstruction more accurately. To further illustrate these points, the following section will present a case study detailing the experiment of the graph convolutional network in MRI reconstruction.

### Graph convolutional network for MRI reconstruction

The “Graph representation of self-similarities” section previously examined how the choice of reference images affects the identification of image patches similar to the target patch. Prior to exploring the core network frameworks outlined in this article, we underscore the critical role of structural similarity between the target image patch and its corresponding similar patches in the context of MRI image reconstruction via Graph Convolutional Networks (GCN). This section aims to substantiate this emphasis through demonstrative verification experiments.

The formulation of a regularized MRI reconstruction framework that incorporates graph convolution as the GCN, can be expressed as follows:

10 $$\mathop {\min }\limits_{x} \lambda \mathop \sum \limits_{j = 1}^{J} \left\| {{\varvec{y}}_{j} - {\varvec{F}}_{u} {\varvec{C}}_{j} {\varvec{x}}} \right\|_{2}^{2} + \left\| {f_{gcn} \left( {{\varvec{x}}_{u} |{\varvec{\theta}}_{gcn} } \right) - {\varvec{x}}} \right\|_{2}^{2} ,$$

where $f_{gcn} \left( \cdot \right)$ symbolizes the neural network model parameterized by ${{\varvec{\uptheta}}}_{gcn}$. Since the k-space data are undersampled, a ground truth image for learning patch similarity directly is unavailable. To address this, we utilize a pre-reconstructed image obtained via SPIRiT [36] to infer patch similarities. These learned similarities align more closely with the optimal similarity compared to those derived from undersampled image, which is clearly illustrated in Fig. 2 in previous section.

**Fig. 3:**
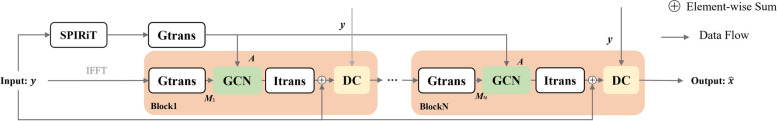
This network block consists of GCN and DC parts, with the blocks cascaded. Graph transformer (Gtrans) and image transformer (Itrans) are the function of image-to-graph and graph-to-image, respectively. The adjacency matrix ***A*** and features ***M*** are obtained from the reconstructed image and the undersampled image respectively

The flowchart of employing GCN in a network to reconstruct MRI images is illustrated in Fig. 3. The Graph transformer (Gtrans) module in Fig. 3 transforms an image into a graph, comprising graph nodes (patches) and graph weights (similarities). The flowed ${\varvec{A}}$ from Gtrans indicates that graph weights flow to the subsequent module, and flowed ${\varvec{M}}_{i}$ ($i = 1, \cdots ,N$) from Gtrans denotes graph nodes (patches) flow into next module. The sampled partial k-space data have been acquired so that network don’t have the necessary to reconstruct. Data Consistency (DC) using the sampled k-space data wisely will enhance the data fidelity [20]:

11 $${\hat{\mathbf{k}}} = \left( {{\varvec{1}}_{H} - {\varvec{H}}} \right) \odot \hat{\user2{k}} + \lambda {\varvec{k}}_{u} ,$$

where ${\hat{\varvec{k}}}$ is the reconstructed k-space data corresponding to reconstructed image. $({\varvec{1}}_{H} - {\varvec{H}})$ strands for the inverse undersampling pattern. $\odot$ represents the multiplication of corresponding elements in the matrix. ${\varvec{k}}_{u}$ denotes the k-space data which is acquired from coils. The acquisition of k-space from the coils is not noise free. Therefore, the $\lambda$ is used to balance the k-space data fidelity between sampled data and the reconstructed k-space data from the network. DC is realized by replacing the k-th predicted data with the original k-space data if it has been sampled. To obtain the forward pass of the layer performing data consistency in k-space:

12 $$f_{dc} \left( {\hat{\varvec{x}},{\varvec{k}}_{u} ,\lambda } \right) = \mathop \sum \limits_{j}^{J} {\varvec{C}}_{j}^{H} {\varvec{F}}^{ - 1} \left( {{\varvec{FC}}_{j} \hat{\varvec{x}} + \lambda {\varvec{k}}_{u} } \right).$$

We set $\lambda$ to a very small value ($\lambda = 1 - 1 \times 10^{ - 6}$) to ensure that the collected data is fully fidelity meanwhile the noise is well suppressed.

**Fig. 4:**
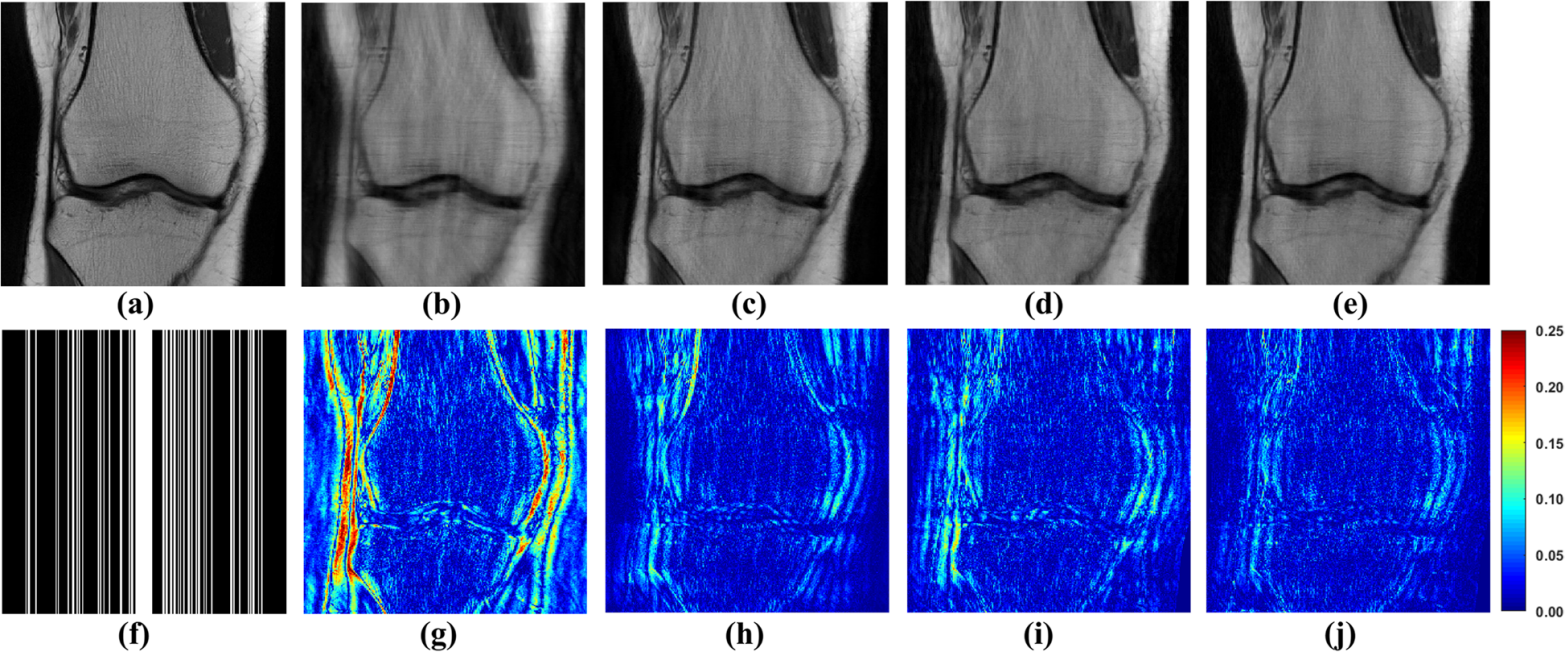
The proposed GCN for MRI reconstructions. **a** Is the fully sampled image. **b** Is the undersampled image. **f** Is the 1D Cartesian undersampling pattern with AF = 4. **c**-**e** Are reconstructed images by SPIRiT, UnGCN, and RecGCN, respectively. **g**-**j** Are the corresponding error maps

The relative $\ell_{2}$ norm error (RLNE) [5] is utilized to compute the reconstruction errors. The RLNE is defined as:

13 $$RLNE = \left\| {{\varvec{x}} - \hat{\varvec{x}}} \right\|_{2} /\left\| {\varvec{x}} \right\|_{2} ,$$

where ${\hat{\varvec{x}}}$ is the reconstructed image and $\varvec{x}$ denotes the fully sampled image. The reconstructed images shown Fig. 4 and RLNE in Table 1 present the benefit of GCN. In which, similarity calculated from undersampled image is referred to as GCN with undersampled similarity (UnGCN), while similarity obtained from, while similarity obtained from a pre-reconstructed image is denoted as GCN with reconstructed image (RecGCN). The number of blocks (filter trainable parameters number is 64 × 36 × 2 × 10) is set to 10.

**Table 1:** Quantitative results (RLNE) of the compared method (Mean ± standard)

Method	AF = 4 (1D Cartesian)		
	SPIRiT	UnGCN	**RecGCN**
RLNE × 100	10.10 ± 1.16	9.80 ± 0.97	**8.55 ± 0.81**

Figure 4 and Table 1 demonstrate that GCN, when equipped with accurately learned similarities, facilitates effective MRI image reconstruction. The outcomes with RecGCN, as depicted in Fig. 4e, highlight superior artifact reduction and edge restoration, underscoring the significance of leveraging non-local information and self-similarity for effective reconstruction of target image patches. Conversely, Fig. 4d illustrates that when similarities are inaccurately determined (stemming from the reliance on undersampled images for similarity weight derivation), the chosen connected blocks can significantly deviate, leading to diminished reconstruction quality.

The validation experiment conducted in this section lays a foundational groundwork for subsequent studies. Future research will concentrate on extracting similarities from images reconstructed using SPIRiT, addressing the inherent difficulties associated with undersampled data in real applications.

### The proposed GCESS for MRI reconstruction

**Fig. 5:**
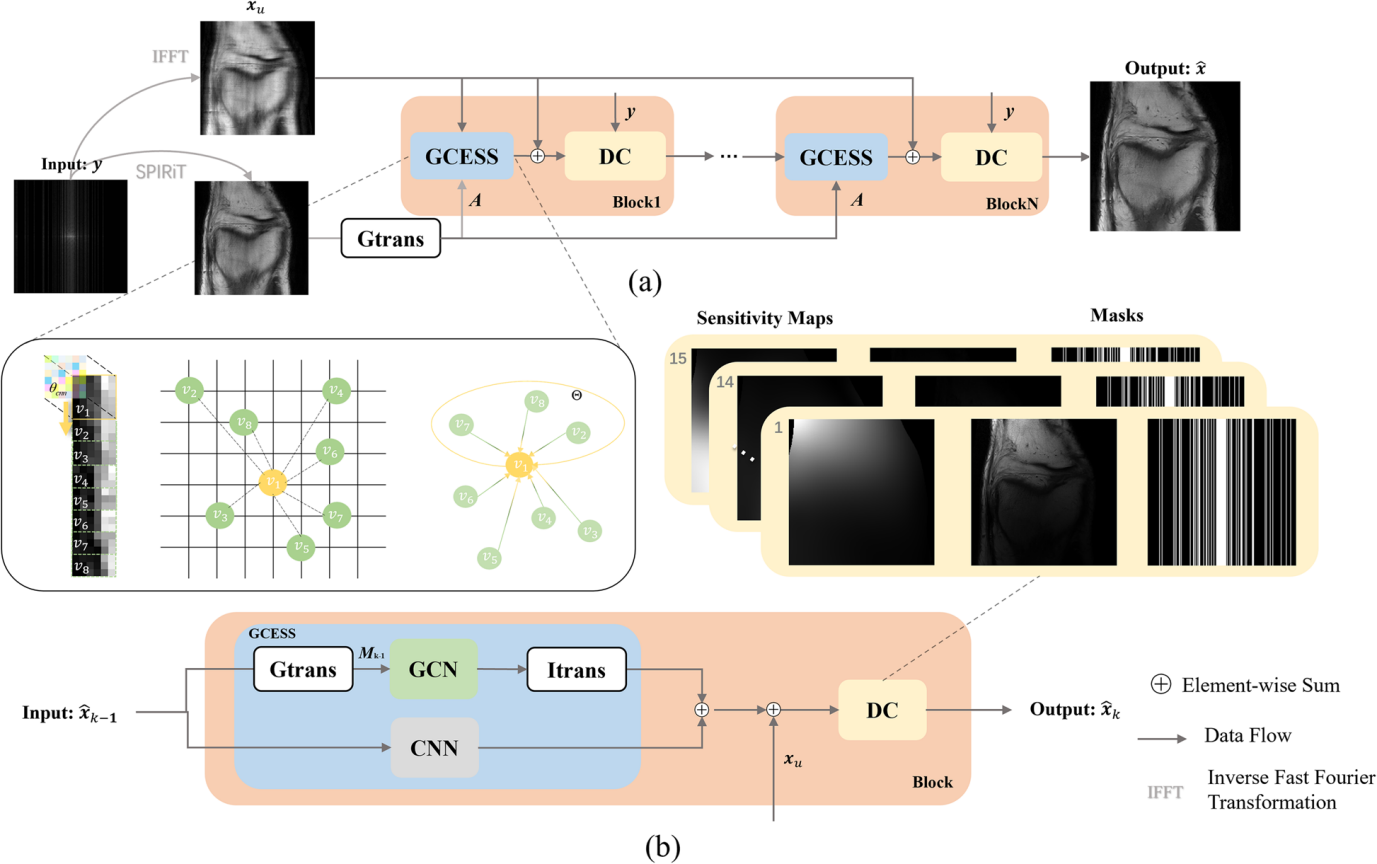
The proposed GCESS for MRI reconstruction. **a** Is the proposed network consisting of GCESS and DC, with blocks cascaded. The graph is learned from SPIRiT pre-reconstructed image. **b** Is a detailed analysis of the main part of the network block

Overlooking local context information within image domain is unwise for MRI reconstructions. It provides essential details about the spatial relationships and texture patterns unique to different regions of the MRI images. This information is pivotal for reconstructing images with high fidelity, ensuring that subtle anatomical structures are accurately represented. Therefore, local information captured by CNNs and non-local information harnessed by GCN are combined to form GCESS network:

14 $$\mathop {\min }\limits_{x} \lambda \mathop \sum \limits_{j = 1}^{J} \left\| {{\varvec{y}}_{j} - {\varvec{F}}_{u} {\varvec{C}}_{j} {\varvec{x}}} \right\|_{2}^{2} + \left\| {f_{gcess} \left( {{\varvec{x}}_{u} |{\varvec{\theta}}_{gcess} } \right) - {\varvec{x}}} \right\|_{2}^{2} ,$$

where $f_{gcess} \left( \cdot \right)$ symbolizes the neural network model parameterized by ${{\varvec{\uptheta}}}_{gcess}$, including parallel implement of GCN and CNNs. The operational flow of our proposed network is illustrated in Fig. 5, where the GCN is synergistically combined with CNNs to constitute GCESS module. The undersampled image ${\varvec{x}}_{u}$ is the input of the integrative network. Before $\varvec{y}$ enters the Gtrans, a SPIRiT-based pre-reconstructed image is obtained to learn similar weight through Gtrans.

Graph convolution leverages non-local similarity information from the adjacency matrix, as illustrated in Fig. 1. This method, combined with CNNs, which capture pixel-level details and broader features, enhances image reconstruction. As depicted in Fig. 5, spatial convolution filtering reconstructs the details of image patches, ensuring comprehensive reconstruction of information across all patches. During reconstruction, graph convolution selectively utilizes patches most similar to the target patch, scattered across the grid image range, to refine the restoration of the target patch. The Itrans put graphs node features back into MRI images canvas to carry out GCN reconstruction, and combine with reconstructed result of CNNs to form GCESS. ResNet [10] incorporates an additional step by adding the input to the neural networks preliminary result of GCESS, following by a DC module. By combining these two powerful mechanisms, GCESS aims to enhance the accuracy and quality of MRI reconstructions, providing a more comprehensive understanding of both local and global contextual information. This holistic approach ensures that the reconstructed images are not only detailed and precise but also maintain a coherent structure that reflects both the immediate and extended spatial relationships inherent in the original MRI data.

## Result

Experiments are implemented in Python 3 using PyTorch as the backend. Training, validation, and testing were performed on a seventh-generation Intel Core i7 processor with 32 GB of RAM and an RTX 3090 GPU (24 GB memory).

### Datasets

This paper leverages two datasets from open repositories: the knee dataset of Variational Network (VN) [18] and the fastMRI [43] brain dataset, both from open repositories. The coil sensitivity maps were estimated from the central k-space region of each slice using ESPIRiT [44].

The public knee dataset provided by VN [18] is utilized in our experiments to assess the performance of our proposed method. This dataset consists of coronal density-weighted k-space data collected from a 2D turbo-spin echo sequence on a 3 T MRI system (Siemens Magnetom Skyra) using 15 coils. It includes data from 20 subjects, with each subject contributing approximately 35 slices. For each subject used for the experiment, the central twenty slices of size $256 \times 256$ were selected. The dataset division was as follows: fourteen subjects (280 slices) were allocated for training, two for validation (40 slices), and the remainder for testing (80 slices).

Additionally, the fastMRI [43] open dataset provides multi-coil T2 weighted k-space data. This dataset encompasses 45 subjects, with around 427 slices in total. Similar to the knee dataset, we selected the central twenty slices of size $320 \times 320$ from each subject for our experiments. The distribution of slices for this dataset was 296 for training, 36 for validation, and the remaining 95 slices were designated for testing.

### Network

As depicted in Fig. 5, the architecture of the network features 10 iterative blocks, each comprising 2 GCN layers and 4 CNN layers, with Batch Normalization (BN) applied to each CNN layer. The CNNs are structured into four layers, with each layer hosting 64 filters of size $3 \times 3$. The Gtrans operator transforms images into graphs, setting the stage for graph node features to serve as inputs for the GCN. Conversely, Itrans acts as the reverse operator to Gtrans, where the output features are transposed back onto the canvas to generate the reconstructed image. The models in our experiment were trained 100 epochs. All filters were initialized by using “normal” initialization [45], and Adam [46] was chosen as the optimizer in the training phase with a learning rate of 0.0015.

The first step in forming the adjacency matrix involves calculating the Gaussian distance to measure the variance between each image patch, a procedure that is notably lengthy. Consequently, updating the adjacency matrix during training becomes a time-intensive process. The time to calculate one adjacency matrix of $256 \times 256$ image each is 4.6 s and each of $320 \times 320$ image is 9.6 s. However, the non-local information in the undersampled parallel MRI images is inaccurate. To address these challenges, we employ SPIRiT as pre-reconstruction technique to refine non-local information extracted from the graph. The reconstructions time of SPIRiT is 15.8 s. The training time of GCESS is 11.2 h while the reconstructing time is 0.14 s (exclude computing adjacency matrix and pre-reconstruction time). The code can be accessed at https://github.com/Qiaoyu-K/GCESS-MRI-master.

### Evaluation criteria

To objectively evaluate the image reconstruction quality of all compared methods in an objective view, we use RLNE [5], structure similarity index measure (SSIM) [47], and the peak signal-to-noise ratio (PSNR) as the quantitative criteria. The RLNE is detailed in Eq. (10).

The SSIM is defined as:

15 $$SSIM = \frac{{\left( {2\mu_{x} \mu_{{\hat{x}}} + C_{1} } \right)\left( {2\sigma_{{x\hat{x}}} + C_{2} } \right)}}{{\left( {\mu_{x}^{2} + \mu_{{\hat{x}}}^{2} + C_{1} } \right)\left( {\sigma_{x}^{2} + \sigma_{{\hat{x}}}^{2} + C_{2} } \right)}},$$

where $\mu_{{x}}$ and $\mu_{{{\hat{x}}}}$ denote the means of ${\varvec{x}}$ and ${\hat{\varvec{x}}}$, $\sigma_{x}$ and $\sigma_{{{\hat {x}}}}$ is the standard deviations of $\varvec {x}$ and ${\hat{\varvec{x}}}$, and $\sigma_{{x\hat{x}}}$ is the covariance of $\varvec{x}$ and ${\hat{\varvec{x}}}$. $C_{1}$, $C_{2}$ is a constant to maintain stability close to zero.

The PSNR is defined as:

16 $$PSNR\left( {dB} \right) = 10 \cdot \log_{10} \left( {\frac{{PQ\left\| {\varvec{x}} \right\|_{\infty } }}{{\left\| {{\varvec{x}} - \hat{\user2{x}}} \right\|_{2} }}} \right),$$

$P$ and $Q$ represent the dimension of the frequency encoding and phase encoding, respectively.

A lower reconstruction error with the lower RLNE signify higher consistencies between reconstructed and fully sampled images. A higher PSNR means better signal-to-noise ratio, and a higher SSIM values indicate better detail preservation and fewer image distortions in the reconstruction.

### Comparison with existing methods

The MRI reconstruction performance of the proposed GCESS model is evaluated against three deep learning methods and one conventional method. The conventional method employed for comparative analysis is SPIRiT [36]. We fine-tuned the parameters of SPIRiT to optimize its performance on our dataset. The testing result shows that it adopted the parameter calibration kernel size $3 \times 3$ and Tikhonov regularization in the calibration was set to be $10^{ - 3}$. The Tikhonov regularization for reconstruction was implemented for $10^{ - 5}$ with SPIRiT, which underwent 30 iterations. The deep learning methods compared include IUNET [48], DCCNN [20] and MoDL [17]. IUNET [48] serves as baseline of MRI image reconstruction. DCCNN represents an early adoption of deep learning in MRI reconstruction, with each iteration comprising 6 CNN layers, following the original publication’s configuration. To ensure fairness, we incorporated a BN layer into each layer of CNNs to enhance network optimization. MoDL [17] is celebrated as a pioneering model-driven deep learning framework in MRI reconstruction, known for reaching performance saturation after approximately 8–10 iterations, each comprising 4 CNN layers with both forward and backward layers containing 64 filters with kernel size of $3 \times 3$. In addition, we added MICCAN [23] and MD-Recon-Net [29] as comparative experiments in additional quantitative comparisons of VN datasets.

**Fig. 6:**
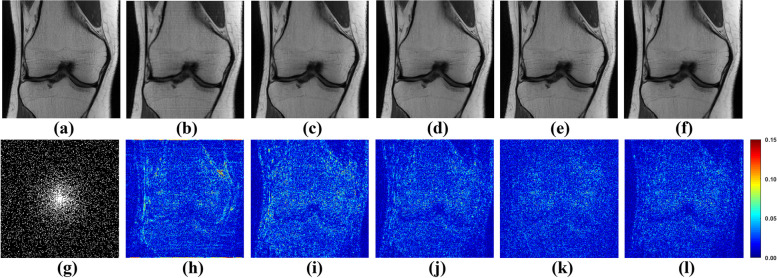
The proposed GCESS network compared with state-of-the-art MRI reconstruction methods. **a** Is the fully sampled image. The experiments correspond to a 2D random sampling with AF = 8 as shown in (**g**). **b**-**f** Are the images of reconstructed results by SPIRiT, IUNET, DCCNN, MoDL, and GCESS, respectively. **h**-**l** are the corresponding error maps. The PSNR of (**b**-**f**) are, 32.51, 29.04, 33.67, 33.99 and 34.42 dB, respectively

**Fig. 7:**
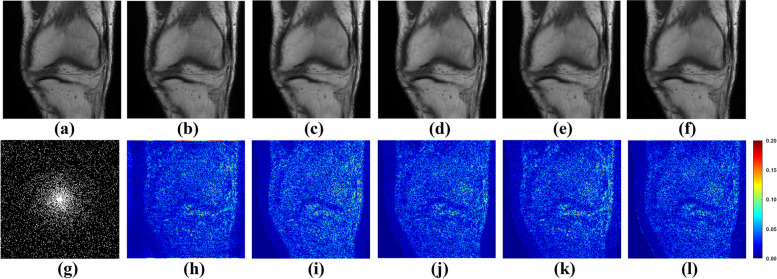
The proposed GCESS network compared with state-of-the-art MRI reconstruction methods. **a** Is the fully sampled image. The experiments correspond to a 2D random sampling with AF = 10 as shown in (**g**). **b**-**f** are the images of reconstructed results by SPIRiT, IUNET, DCCNN, MoDL, and GCESS, respectively. **h**-**l** are the corresponding error maps. The PSNR of (b)-(f) are 33.46, 32.79, 33.95, 33.63 and 34.29 dB, respectively

**Fig. 8:**
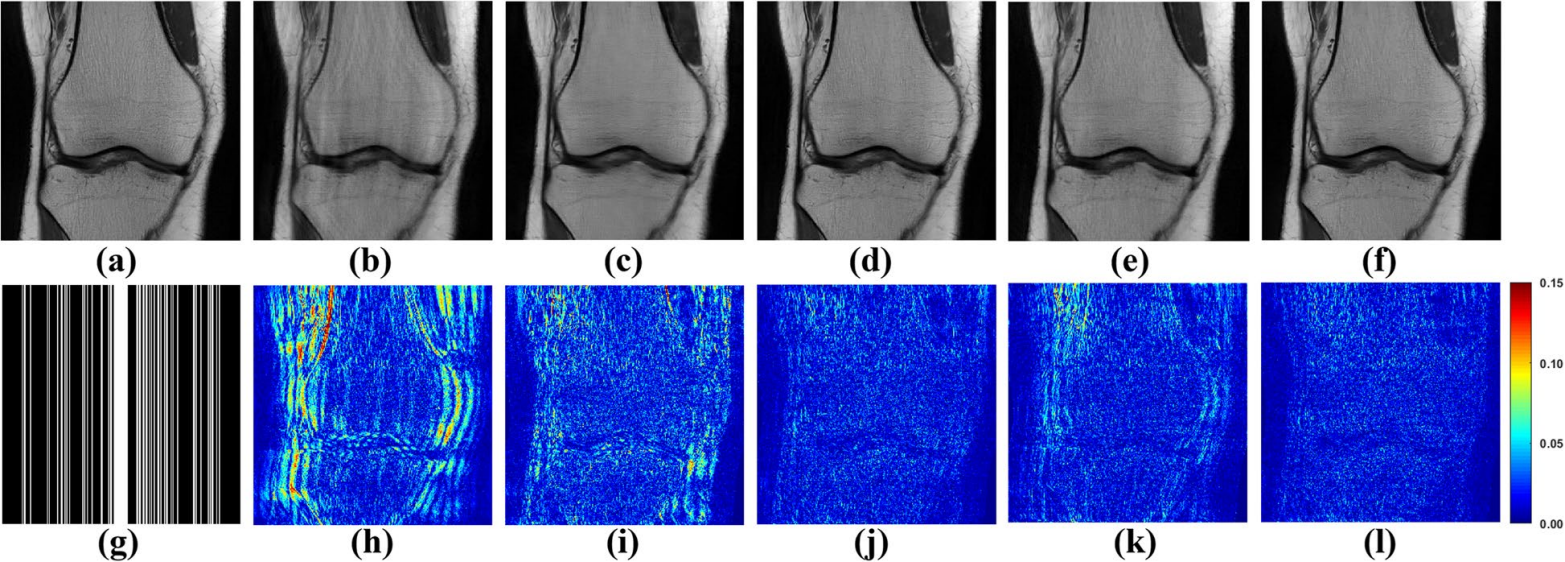
The proposed GCESS network compared with state-of-the-art MRI reconstruction methods. **a** Is the fully sampled image. The experiments correspond to a 1D Cartesian sampling with AF = 4 as shown in (**g**). **b**-**f** Are the images of reconstructed results by SPIRiT, IUNET, DCCNN, MoDL, and GCESS, respectively. **h**-**l** are the corresponding error maps. The PSNR of (**b**-**f**) are 28.68, 30.73, 34.05, 32.83 and 34.69 dB, respectively

**Fig. 9:**
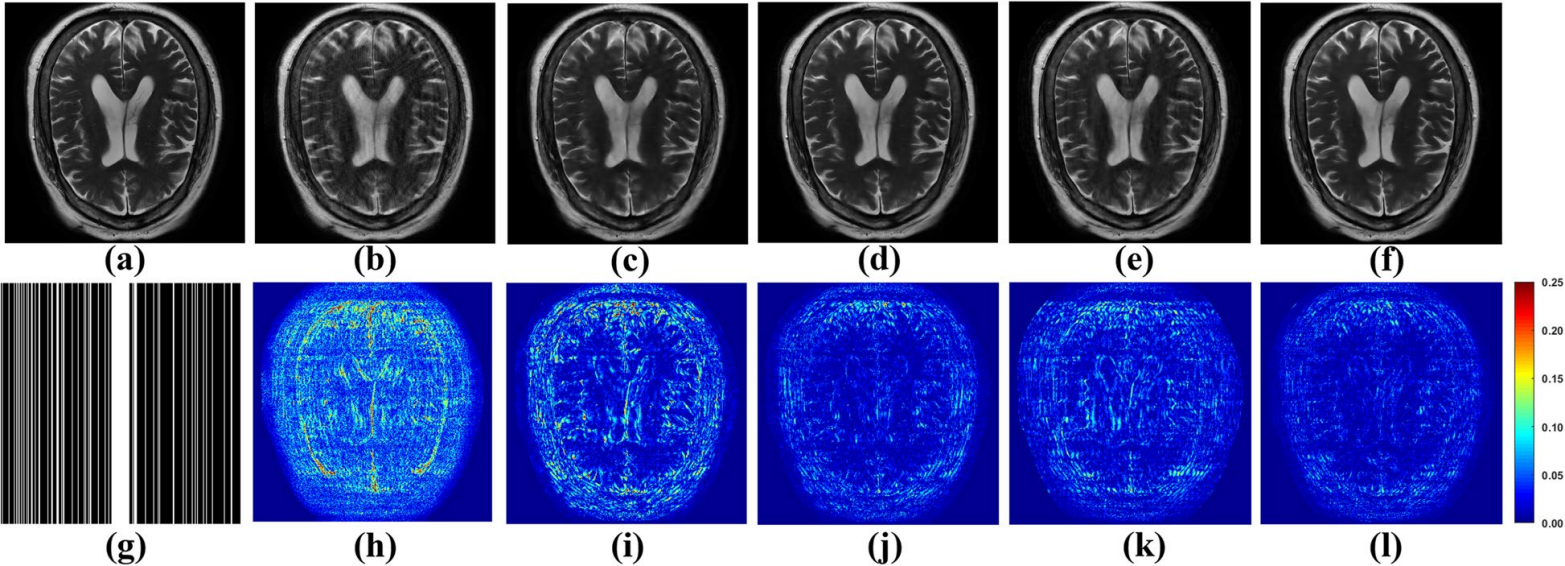
The proposed GCESS network compared with state-of-the-art MRI reconstruction methods. **a** Is the fully sampled image. The experiments correspond to a 1D Cartesian sampling with AF = 4 as shown in (**g**). **b**-**f** Are the images of reconstructed results by SPIRiT, IUNET, DCCNN, MoDL, and GCESS, respectively. **h**-**l** Are the corresponding error maps. The PSNR of (**b**-**f**) are 28.67, 29.44, 33.33, 32.15 and 34.61 dB, respectively

To appraise the efficacy of the proposed method, both one-dimensional (1D) Cartesian undersampling pattern and two-dimensional (2D) random undersampling were adopted. The reconstructed images and corresponding error maps of the compared methods with different acceleration factors are presented in Figs. 6, 7, 8 and 9. From the reconstruction errors in Figs. 6, 7, 8 and 9, the SPIRiT and the IUNET have obvious artifacts as illustrated in Figs. 6, 7, 8 and 9b. MoDL outperforms DCCNN in artifacts suppression, whereas GCESS shows the highest efficacy in minimizing artifacts. The comparative analysis of Figs. 6c-d and 7c-d illustrates that MoDL’s reconstruction quality declines more rapidly than GCESS’s with increased acceleration factors.

Table 2 consolidates the average numerical performance, along with standard deviations, for the testing knee datasets across the evaluated methods, showcasing quantitative metrics for both 2D random undersampling with acceleration factors (AF) of 8 and 10, and 1D Cartesian undersampling with AF of 4. The GCESS model’s superior reconstruction quality is evidenced by its leading performance metrics in PSNR, SSIM, and RLNE values, underlining its effectiveness in MRI reconstruction.

**Table 2:** Quantitative results (RLNE, PSNR, and SSIM) of the compared method (Mean ± standard)

Pattern	Method	RLNE × 100	SSIM × 100	PSNR
Knee dataset				
1D Cartesian AF = 4	SPIRiT	10.10 ± 1.16	81.75 ± 1.97	28.32 ± 0.98
	IUNET	9.02 ± 1.29	80.88 ± 3.66	29.36 ± 1.00
	DCCNN	5.86 ± 0.94	88.20 ± 1.87	33.14 ± 1.36
	MoDL	7.01 ± 0.97	85.81 ± 3.15	31.56 ± 1.19
	MICCAN	19.49 ± 4.23	76.65 ± 3.05	23.44 ± 2.09
	MD-Recon-Net	8.68 ± 1.72	81.49 ± 4.40	29.79 ± 1.97
	**GCESS**	**5.10 ± 0.88**	**89.94 ± 2.39**	**34.19 ± 1.52**
2D Random AF = 8	SPIRiT	6.32 ± 0.69	83.13 ± 1.55	32.41 ± 1.13
	IUNET	9.44 ± 1.14	74.36 ± 5.67	28.95 ± 1.24
	DCCNN	5.21 ± 0.80	87.46 ± 3.03	34.16 ± 1.80
	MoDL	4.87 ± 0.75	86.78 ± 3.67	34.86 ± 1.90
	MICCAN	11.34 ± 3.53	77.90 ± 4.34	28.33 ± 2.09
	MD-Recon-Net	10.56 ± 1.60	71.16 ± 4.80	28.01 ± 1.36
	**GCESS**	**4.69 ± 0.76**	**88.12 ± 3.34**	**35.09 ± 1.93**
2D Random AF = 10	SPIRiT	6.90 ± 0.67	80.45 ± 2. 01	31.65 ± 1.10
	IUNET	9.81 ± 1.29	72.38 ± 6.35	28.63 ± 1.31
	DCCNN	5.67 ± 0.88	85.06 ± 3.77	33.42 ± 1.81
	MoDL	6.13 ± 0.78	83.05 ± 3.90	32.70 ± 1.57
	MICCAN	11.11 ± 3.10	78.15 ± 5.42	28.42 ± 2.91
	MD-Recon-Net	10.86 ± 1.65	70.57 ± 5.10	27.76 ± 1.31
	**GCESS**	**5.22 ± 0.85**	**85.39 ± 4.15**	**34.16 ± 1.94**
Brain dataset				
1D Cartesian AF = 4	SPIRiT	14.90 ± 1.93	85.24 ± 2.41	29.83 ± 0.64
	IUNET	13.06 ± 1.16	90.54 ± 1.69	30.94 ± 0.91
	DCCNN	8.48 ± 1.14	94.62 ± 1.29	34.73 ± 1.02
	MoDL	12.17 ± 5.20	90.79 ± 4.26	32.01 ± 2.82
	**GCESS**	**7.57 ± 1.17**	**95.36 ± 1.27**	**35.73 ± 1.14**

The lowest RLNE, highest PSNR and SSIM values are bold faced

### Ablation studies

To verify the effectiveness of the proposed integrated network simultaneously extract both non-local and local information, we carried out ablation studies. These studies were designed to assess the impact of various critical components within the proposed network architecture.

**Fig. 10:**
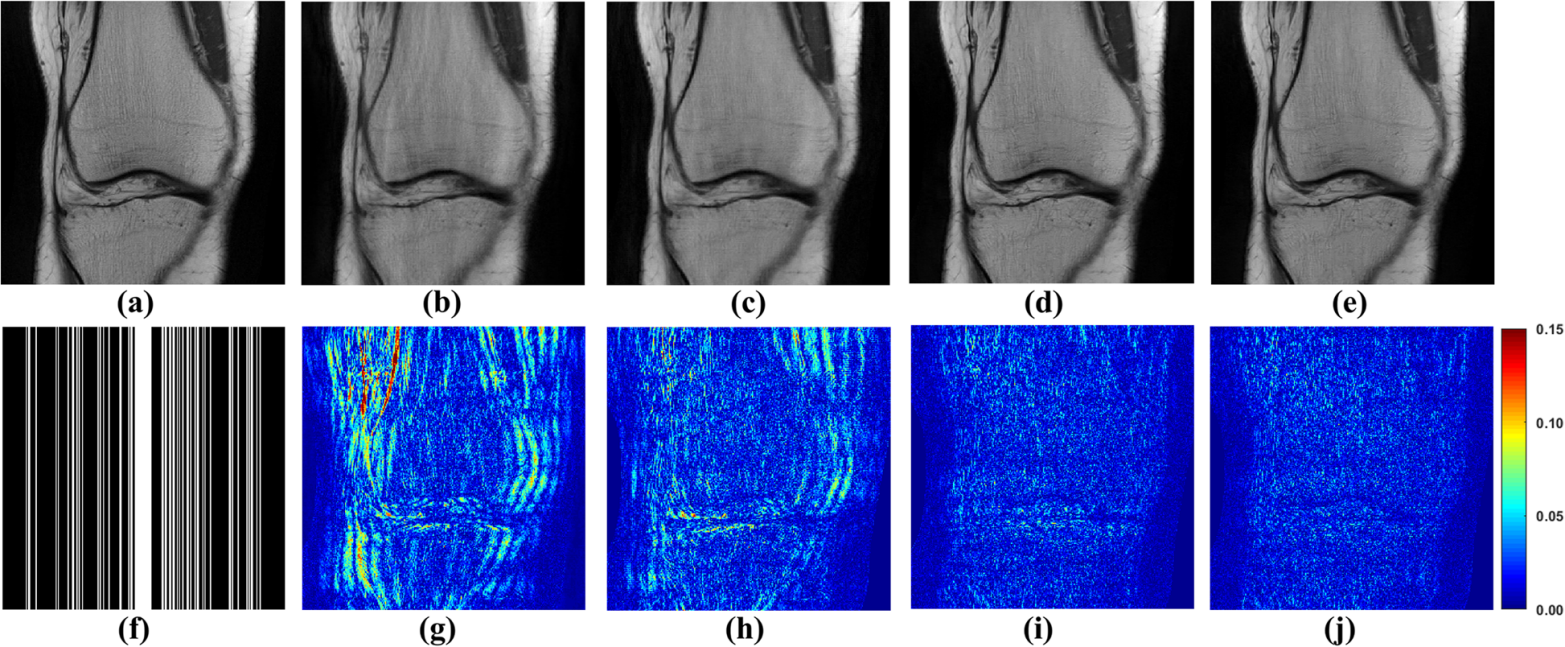
Ablation studies results of the proposed GCESS network. **a** Is the fully sampled image. **f** Is the 1D Cartesian undersampling pattern with AF = 4. **b**-**e** Are the images of reconstructed results by SPIRiT, GCN, CNNs, and GCESS, respectively. **g**-**j** Are the corresponding error maps. The PSNR of (**b**-**e**) are 29.63,31.69, 34.51 and 34.95 dB, respectively

Specifically, we eliminated the graph convolution from GCESS, resulting in a model that relies solely on local information (CNNs). Conversely, by removing the CNNs component from GCESS, we isolated the GCN component (the same as GCN in “Graph convolution with enhanced self-similarity” section) which simply rely on non-local information. Figure 10 showcases the reconstructed result with 1D Cartesian undersampling pattern with AF of 4. In these results, the GCN model demonstrates strong artifact suppression capabilities. Compared to CNNs, GCESS achieves a notable reduction in global error, showcasing the advantage of integrating both non-local and local information for enhanced quantitative outcomes. Table 3 summarizes the quantitative results of the entire test dataset with 1D Cartesian undersampling pattern with AF of 4, highlighting GCESS’s superior performance metrics compared to the standalone CNNs and GCN models across all evaluated parameters.

**Table 3:** Quantitative results (RLNE, PSNR, and SSIM) of the compared method (Mean ± standard)

Method	RLNE × 100	SSIM × 100	PSNR
GCN	8.55 ± 0.81	84.39 ± 2.82	29.78 ± 0.91
CNNs	5.72 ± 1.13	89.21 ± 2.68	33.39 ± 1.69
**GCESS**	**5.19 ± 0.88**	**89.94 ± 2.39**	**34.19 ± 1.52**

The lowest RLNE, highest PSNR and SSIM values are bold faced

In summary, local information ensures the fidelity of reconstructed images in representing fine details and textures, which are crucial for diagnostic accuracy. Non-local information facilitates the identification of repeating patterns and structures across the image, allowing for a more robust reconstruction by filling in gaps that local information alone might not address, especially in edge regions.

## Discussion

This work focus on the development and application GCESS network for MRI reconstructions. The emphasis on non-local information in MRI image reconstruction stems from its potential to capture broader, contextually relevant patterns across the entire image, which local information alone might miss. Additionally, Non-local information can help in identifying and leveraging the inherent redundancy within MRI images, such as similar structural patterns across different regions, which is crucial for the effective reconstruction of artifacts. Meanwhile, local information provides high-resolution details and fine-grained features essential for accurately capturing the intricacies of structures. Hence, the architectural design of this network not only inherits the extracting local information advantage of CNNs but also utilizes GCN to make full use of non-local information to eliminate artifacts. Traditional local spatial convolutional directly operation on image, while we construct MRI image into graph as the input of GCN to represent the non-local self-similarity information of the image. The non-local information in the graph constructed similarity relations between image patches which does not adjoin in the grid-like data but shares lots of structure information through the connected edge of graph. In GCN-based training, MRI reconstructions are regarded as node (patch) reconstruction.

Our method also has limitations. The first step of constructing the graph is finding the eight most similar image patches for each patch. This process must calculate the Gaussian distance as the similarity between patches (time-consuming 8.6 s). Although we have tried numerous sorts of methods like stacking image patches or using GPU to speed up computation, the problem of time-consuming still exists. Because of the above time reasons, it is difficult to update the graph after every epoch of the training process. Thus, we use SPIRiT as our pre-reconstruct method to fix non-local information extracted from image. To meet the time requirement of clinical practice, a more computationally efficient method or an embedded graph learning network is to be further developed. This will be considered in our next work.

## Conclusions

In this work, Graph Convolution network with Enhanced Self-Similarity (GCESS) is introduced which combine local information and non-local self-similarity information for MRI reconstruction. Local information is harnessed through the traditional means of a convolutional neural network. The non-local self-similarity is captured via graph representation and processed through graph convolution. As the network undergoes training, self-similarity is accentuated, and the graph convolution filters are updated. This enhanced self-similarity information subsequently directs the reconstruction process, leveraging the non-local information conveyed through the graph edges. This methodology enriches the target patch with additional non-local similarity information, facilitating superior image’s artifact suppression and edge preservation. Experimental in vivo datasets demonstrate that the proposed network achieves superior reconstruction outcomes compared to existing state-of-the-art methods. Specifically, our approach yields reconstructions with reduced errors and enhanced detail and fine structure preservation.

## Data Availability

The data used in this paper are public datasets.

## Abbreviations

MRI: Magnetic resonance imaging
GCESS: Graph Convolution network with Enhanced Self-Similarity
GCN: Graph Convolutional Network
CNNs: Convolutional Neural Networks
Gtrans: Graph transformer
DC: Data Consistency
UnGCN: GCN with undersampled similarity
RecGCN: GCN with reconstructed image
RLNE: Relative $\ell_{2}$ Norm Error
SSIM: Structure Similarity Index Measure
PSNR: Peak Signal-to-Noise Ratio
